# Synergistic Growth and Metabolic Interactions of *Kluyveromyces marxianus* and *Lactococcus lactis* in Rose-Aroma Fermented Milk Revealed by Integrated Flavoromics and Metabolomics

**DOI:** 10.3390/metabo16040235

**Published:** 2026-03-31

**Authors:** Jiawen Liu, Ziyan Yue, Yuyao He, Xinchi Jiang, Hong Zeng, Yanbo Wang

**Affiliations:** 1 School of Food and Health, Beijing Technology and Business University, Beijing 100048, China; 19932631278@163.com (J.L.); 15525794820@163.com (Z.Y.); 13875845861@163.com (Y.H.); 18515391598@163.com (X.J.); 2 Key Laboratory of Geriatric Nutrition and Health, Beijing Technology and Business University, Beijing 100048, China

**Keywords:** fermented milk, *Kluyveromyces marxianus*, *Lactococcus lactis*, volatile organic compounds, odor activity, metabolomics, rose aroma

## Abstract

Background/Objectives: Fermented dairy products typically rely on the starter culture of lactic acid bacteria (LAB), resulting in relatively homogeneous flavor profiles and loss of flavor diversity. Methods: This study employed flavoromics and untargeted metabolomics to evaluate the flavor modulation effects of rose-aroma producing *Kluyveromyces marxianus* co-cultured with *Lactococcus lactis* on the fermented milk. Results: In the co-culture group, *K. marxianus* (KM) was able to promote the growth of *L. lactis* (LC). KMLC co-culture exhibited superior sensory evaluation and flavor characteristics and a more pronounced rose aroma compared with the monoculture groups and the commercial fermented milk groups. During the fermentation of the KMLC group, 15 key VOCs were identified through OAV analysis, while 37 key metabolites were identified based on variable importance in projection (VIP) > 1 and probability value (*p*) < 0.05. Spearman correlation analysis revealed a significant correlation between key metabolites and key VOCs. Furthermore, key metabolites played a crucial role in the formation of floral and fruity flavors by participating in metabolic pathways such as citrate metabolism, nucleotide metabolism, and phosphate metabolism. Conclusions: This study demonstrated that *K. marxianus* and *L. lactis* co-culture could significantly enhance the rose aroma of fermented milk, providing solid evidence for flavor innovation in fermented milk through the application of *K. marxianus*-composite starter cultures.

## 1. Introduction

Fermented milk products are valued for their refreshing flavor, high nutritional content, and diverse microbial resources [1]. However, traditional fermented milk products typically rely solely on lactic acid bacteria (LAB) as starter cultures [2], bringing significant challenges due to a lack of flavor complexity. While effective in fermentation, such starter cultures can lead to the excessive hydrolysis of lipids and proteins, causing overly acidic flavors and insufficient flavor depth [3]. These shortcomings not only diminish the consumer eating experience but also hinder the market competitiveness of traditional fermented milk, thereby pointing out a critical need to develop fermented milk with a unique, impressive, and enhanced flavor experience.

Recently, many studies have shown that the inclusion of yeast in fermentation can promote the formation of a unique flavor of fermented dairy products [4]. *Kluyveromyces marxianus* (*K. marxianus*) can produce fragrant compounds such as 2-phenylethanol and ethyl acetate, which can bring fermented milk with a buttery, cheesy, rose aroma, and a slightly sweet taste [5,6]. *K. marxianus* utilizes aromatic amino acids generated during metabolism to synthesize higher alcohols, like phenylethanol, and esters such as ethyl phenylacetate, which together impart a unique floral and fruity aroma to the products [7]. For instance, Yang et al. [8] demonstrated that *K. marxianus* YE27 effectively reduced grassy aromas and enhanced floral and fruity aromas during black tea fermentation, while significantly increasing the content of ester compounds with fruity aromas. Furthermore, the exogenous addition of L-phenylalanine to the fermentation system enables *K. marxianus* to produce aromatic compounds, such as 2-phenylethanol, via the Ehrlich pathway, thereby enhancing the rose aroma of the system [9]. Roy et al. indicated that the addition of L-phenylalanine to the fermentation system of sweet lemon peel and sugarcane bagasse significantly increased the concentrations of 2-phenylethanol and ethyl 2-phenylethanoate, resulting in a pronounced rose aroma [10].

Due to its superior ability to produce flavor compounds, *K. marxianus* has been considered as an adjunct flavor culture. There are many yeast–LAB products on the market in Western countries, some marketed as Kefir [11]. When co-cultured with LAB, the Ehrlich pathway and other amino acid metabolic pathways are significantly activated, catalyzing the conversion of phenylalanine into floral and fruity higher alcohols [12]. Additionally, previous studies demonstrated that *K. marxianus* improves fermented milk flavor by promoting LAB growth and regulating amino acid and glycerophospholipid metabolism, thereby increasing ester, acid, ketone, and alcohol production [13,14]. Although the synthesis of aromatic compounds by *K. marxianus* can be enhanced under conditions of exogenous amino acid supplementation or co-culture with LAB, the combined effect of these two approaches and their impact on the enhancement of aroma production in fermented milk remains unclear. Furthermore, the biological mechanisms underlying aroma formation in this co-culture system, including the associated metabolic pathways and microbial interactions, require further elucidation.

This study aims to investigate the biosynthetic mechanisms of key flavor compounds of *K. marxianus* and *Lactococcus lactis* (*L. lactis*) fermented milk with exogenous phenylalanine supplementation. Exogenous phenylalanine was supplemented to optimize fermentation conditions, whereby the aroma of fermented milk was enhanced via activation of the Ehrlich pathway and other aroma-producing metabolic pathways. The growth characteristics of the microorganisms in monoculture and co-culture systems were first analyzed. Commercially available rose-aroma fermented milk and plain fermented milk were selected as reference products. Sensory evaluation and flavor analysis were performed among the monoculture, co-culture, and commercial fermented milk groups, confirming that the co-cultured group possessed a unique and highly recognized rose aroma. Through flavoromics and untargeted metabolomics analysis, the dynamic changes in key volatile organic compounds (VOCs) and key metabolites during the fermentation process of the KMLC group were further investigated, elucidating the formation mechanism of the characteristic rose flavor. Additionally, the study explored the relationships between core functional microorganisms, key VOCs, and key flavor metabolites. The findings provide flavor-chemistry and microbial basis for the development of natural rose-aroma fermented milk.

## 2. Materials and Methods

### 2.1. Materials

The two strains involved in this experiment were *Lactococcus lactis* subspecies *lactis* (CICC B6246) and *Kluyveromyces marxianus* (CICC 1953), which were obtained in lyophilized form from the China Center of Industrial Culture Collection (CICC). *L. lactis* and *K. marxianus* were stored in Man Rogosa and Sharpe (MRS) medium (Land Bridge, China) and Yeast Extract Peptone Dextrose (YPD) medium (Solarbio, China), respectively, both of which contained 25% glycerol and were maintained at −80 °C. *L. lactis* was cultured in MRS liquid medium at 37 °C for 24 h, while *K. marxianus* was cultured in YPD medium at 28 °C for 24 h. Two consecutive cycles of activation and subculturing were performed for both strains. After cultivation, the microbial cells were harvested by centrifugation at 8000× *g* for 10 min at 4 °C and resuspended in sterile normal saline (0.9% sodium chloride). Whole milk powder was sourced from Fonterra (Auckland, New Zealand), and food-grade L-phenylalanine, supplemented as the precursor of 2-phenylethanol, was obtained from Hebei Huayang Biotechnology Co., Ltd. (Yuwanbang), Hengshui, Hebei, China. For the control group, two commercial fermented milk products were purchased from the local Hema Fresh supermarket: Jian’ai sucrose-free plain fermented milk (C-PY) and Europe-Asia Dali Ranch flower-flavored fermented milk (C-RY). The methanol, acetonitrile, dichloromethane, and 2-methyl-3-heptanone, all of analytical grade, were procured from Aladdin (Shanghai, China). The natamycin and clindamycin, both of analytical grade, were procured from Aladdin Ltd., Shanghai, China.

### 2.2. Preparation of Rose-Aroma Fermented Milk Samples

The reconstituted milk was prepared using 11.5% (*w*/*w*) whole milk powder and 0.2% (*w*/*w*) phenylalanine. The selection of optimal processing parameters for fermented milk flavor was detailed in Tables S1–S3. Briefly, whole milk powder and phenylalanine were dissolved in sterile water, and the mixture was subjected to homogenization (Duoning AH-NANO, Shanghai, China). Subsequently, the reconstituted milk was heated to 95 °C and maintained at this temperature for 5 min. After being cooled to 36 °C, starter cultures were inoculated. For the KM batch, *K. marxianus* was inoculated at 4% (*v*/*v*) with an initial viable cell density of 1 × 10^5^ CFU/mL. For the LC batch, *L. lactis* was inoculated at 4% (*v*/*v*) with an initial viable cell density of 1 × 10^7^ CFU/mL. For the KMLC batch, *L. lactis* and *K. marxianus* were co-inoculated at 4% (*v*/*v*) each, with initial viable cell densities of 1 × 10^7^ CFU/mL and 1 × 10^5^ CFU/mL, respectively. Fermentation was carried out under sealed, static conditions at 36 °C without agitation. Samples from the KM, LC, and KMLC batches were collected after 8 h of fermentation; one portion was stored at 4 °C for sensory evaluation alongside two commercial fermented milk products, while another portion was stored at −80 °C for subsequent flavoromics analysis. During the fermentation process, KMLC samples were additionally collected at 0, 2, 3, 4, 5, 6, and 8 h and stored at −80 °C for subsequent amplicon sequencing, flavoromics, and metabolomics analysis.

### 2.3. Quantification of Microbial Growth Kinetics

#### 2.3.1. Changes in Yeast, LAB, and pH in Fermented Milk

At various fermentation time points, fermented milk samples were collected from the KMLC, LC, and KM groups at one-hour intervals for pH measurement and viable cell counting. The entire sampling procedure was conducted under aseptic conditions in a laminar flow hood. Viable cell numbers were determined by the plate counting method, and pH was measured using a pH meter (Mettler Toledo Plus, Zurich, Switzerland). MRS agar was supplemented with 0.5 g/L natamycin to inhibit the growth of *K. marxianus* at 37 °C for 48 h. YPD agar was supplemented with 0.1 g/L clindamycin to inhibit the growth of *L. lactis* at 28 °C for 48 h [13].

#### 2.3.2. Amplicon Sequencing

##### 16S Amplicon Sequencing and Internal Transcribed Spacer Sequencing

Amplicon sequencing was performed using a method described by Chen et al. [15], with slight modifications. Total genomic DNA of the microbial community was extracted from the KMLC group samples using the FastPure Soil DNA Isolation Kit (Magnetic bead) (MJYH, Shanghai, China) according to the manufacturer’s instructions. The quality of the extracted genomic DNA was assessed by 1% agarose gel electrophoresis, and its concentration and purity were measured with a NanoDrop2000 spectrophotometer (Thermo Fisher Scientific, Waltham, MA, USA). The V3-V4 hypervariable region of the bacterial 16S ribosomal RNA (rRNA) gene amplicon sequencing was amplified using the primer pair 338F (5′-ACTCCTACGGGAGGCAGCAG-3′) and 806R (5′-GGACTACHVGGGTWTCTAAT-3′) for 16S rRNA gene amplicon sequencing. The fungal internal transcribed spacer (ITS)1 region was amplified using the primer pair ITS1F (5′-CTTGGTCATTTAGAGGAAGTAA-3′) and ITS2R (5′-GCTGCGTTCTTCATCGATGC-3′) for ITS amplicon sequencing. PCR reactions were performed in a total volume of 20 μL containing 4 μL of 5× TransStart FastPfu buffer (TransGen Biotech, Beijing, China), 2 μL of 2.5 mM dNTPs, 0.8 μL of each primer (5 μM), 0.4 μL of TransStart FastPfu DNA polymerase, and 10 ng of template DNA. The amplification program consisted of an initial denaturation at 95 °C for 3 min, followed by 27 cycles of denaturation at 95 °C for 30 s, annealing at 55 °C for 30 s, and extension at 72 °C for 30 s, with a final extension at 72 °C for 10 min. Purified amplicons were pooled in equimolar amounts and sequenced on an Illumina NextSeq2000 platform (Illumina, San Diego, CA, USA. Control Software v4.0) according to standard protocols of Majorbio Bio-Pharm Technology Co., Ltd. (Shanghai, China).

The acquired raw 16S rRNA and ITS sequence data were processed using fastp (version 0.20.0) for quality control and FLASH (version 1.2.7) for merging paired-end reads. Operational taxonomic units (OTUs) were clustered at 97% similarity using UPARSE (version 11.0.667). The ribosomal database project (RDP) classifier was used to assign 16S rRNA and ITS gene sequences to taxonomic groups based on the SILVA database (version 138.2) and the UNITE fungal ITS database (version 9.0), respectively [16,17,18].

##### Fluorescence Quantification

Quantitative PCR (qPCR) was employed as a proxy for bacterial and fungal abundance estimation [19]. qPCR was performed using SYBR Green I detection (ChamQ SYBR Color qPCR Master Mix (2×), Vazyme, Nanjing, China) on an ABI 7300 Real-Time PCR system (Applied Biosystems, Waltham, MA, USA) [20]. Bacterial 16S rRNA genes were amplified using primers 338F (5′-ACTCCTACGGGAGGCAGCAG-3′) and 806R (5′-GGACTACHVGGGTWTCTAAT-3′), while fungal ITS regions were amplified using primers ITS1F (5′-CTTGGTCATTTAGAGGAAGTAA-3′) and ITS2R (5′-GCTGCGTTCTTCATCGATGC-3′).

### 2.4. Sensory Evaluation

Sensory evaluation of five fermented milk samples (LC, KM, KMLC, C-PY, C-RY) was conducted by quantitative descriptive analysis (QDA) [21]. QDA was employed as a sensory evaluation technique in which a trained panel quantitatively analyzed and described the sensory attributes of the product. A comprehensive assessment of the intensity of each attribute was conducted, thereby integrating qualitative and quantitative analyses [22]. An 8-member expert panel (4 males and 4 females, aged 20–30 years) from Beijing Technology and Business University, non-smokers, free of rhinitis, with more than two years dairy sensory experience, was trained to recognize aroma attributes, each panel member (in an environment isolated from external communication) smelled all fermented milk samples and recorded odor descriptors based on their sensory experience. Subsequently, the final odor attributes (fermented, butter, cream, milky, fruity, rose, cheese) were selected by combining the results from existing research and the descriptors provided by the panelists [21,23,24,25]. Specific details regarding the sensory evaluation were provided in Table S4. Three individual samples (10–15 mL each) were prepared in transparent tasting cups for each group and labeled with randomized three-digit codes. Samples were equilibrated to room temperature, aroma intensity was rated on a 0–9 scale (0 = absent, 7 = reference, 9 > reference) with 30 s pauses and coffee-bean palate reset. Ethical approval was obtained from the Scientific Research Ethics Committee of Beijing Technology and Business University under the approval number 2025-128, and each panelist signed an informed consent statement before the sensory evaluation.

### 2.5. Flavoromics Analysis

Volatile organic compounds present in five groups of fermented milk were extracted by Solid-Phase Microextraction Arrow (SPME-Arrow) (Shimadzu Smart SPME Arrow 1.10 mm: DVB/C-WR/PDMS, Kyoto, Japan). For each sample, 5.00 g was accurately transferred into a 20 mL threaded sample vial and vortex-mixed. Subsequently, 1 µL of 2-methyl-3-heptanone (0.816 mg/mL) was added as an internal standard. The SPME-Arrow fiber was inserted into the injection port in splitless mode at 250 °C and desorbed for 5 min. The vial was then incubated in a water bath at 45 °C for 30 min, followed by exposure of the SPME-Arrow extraction fiber to the headspace at 45 °C for a 30 min extraction.

Gas Chromatography–Mass Spectrometry (GC-MS) analysis was performed on a GCMS-TQ8050 NX system (Shimadzu Corporation, Kyoto, Japan) coupled with the Shimadzu Smart Aroma Database. An SH-polar wax capillary column (60 m × 0.25 mm, 0.25 µm) was employed. The column temperature was initially maintained at 40 °C for 5 min, then ramped to 200 °C at 3 °C/min. Prior to sampling, the fiber was conditioned in the injection port for 5 min to remove potential contaminants. Mass spectrometric detection was conducted using electron impact ionization at 70 eV, with an ion source temperature of 200 °C and a mass scan range of 35–600 m/z [26].

The detected volatile compounds were identified by matching their mass spectra against the NIST 20 standard reference database (https://chemdata.nist.gov/, accessed on 20 October 2025), and only those with match factors exceeding 80% were retained. The retention index (RI) of each volatile substance was calculated from n-alkanes (C7–C40). Quantification of the volatile compounds was performed using the internal standard method, based on the peak area ratio of each compound to that of the internal standard.

### 2.6. Metabolomics Analysis

The KMLC sample (0.4 g) was slowly thawed at 4 °C, mixed with 4 mL of the extraction solvent, vortexed, and subjected to low-temperature ultrasonication for 30 min. The extraction solvent for metabolites was prepared by mixing methanol, acetonitrile, and water at a volume ratio of 2:2:1 (*v*/*v*/*v*). The mixture was then kept at −20 °C for 10 min and subsequently centrifuged at 4 °C and 14,000× *g* for 20 min. The supernatant was collected and dried under vacuum. For mass spectrometric analysis, the dried residue was reconstituted in 100 μL of acetonitrile/water (1:1, *v*/*v*), vortexed, and centrifuged at 4 °C and 1400× *g* for 15 min. The resulting supernatant was transferred for injection analysis [27].

Sample analysis was performed on an ultra-high performance liquid chromatography system (1290 Infinity LC, Agilent Technologies, Santa Clara, CA, USA) coupled with a quadrupole time-of-flight mass spectrometer (AB Sciex TripleTOF 6600, SCIEX, Framingham, MA, USA). Chromatographic separation was achieved on a 2.1 mm × 100 mm ACQUITY UPLC BEH Amide column (1.7 μm particle size; Waters, Wexford, Ireland). The mobile phase was composed of solvent A (25 mmol/L ammonium acetate and 25 mmol/L ammonia in water) and solvent B (acetonitrile). The gradient elution program was set as follows, 95% B was maintained for 0.5 min, then linearly decreased to 65% B over 6.5 min, followed by a reduction to 40% B in 1 min and held for 1 min. Subsequently, the proportion of B was increased to 95% within 0.1 min, followed by a 3 min re-equilibration period. The electrospray ionization source was operated with a spray voltage of ±5500 V in both positive and negative ion modes and an ion source temperature of 600 °C. The mass spectrometry conditions were set as follows: the mass scan range was 60–1000 Da for MS1 and 25–1000 Da for MS2, with accumulation times of 0.20 s and 0.05 s, respectively [28].

The raw mass spectrometry data were converted to MzXML format using ProteoWizard MSConvert software (version 3.0.24012). Peak detection and alignment were subsequently performed using the XCMS package (version 3.7.1) in R. A collection of metabolite annotation algorithms was employed to label isotopes and adducts. Metabolite identification was achieved by matching the accurate mass-to-charge ratios (mass error < 10 ppm) and MS/MS spectra against a laboratory-built database established using authentic standards.

### 2.7. Data Analysis

All measurements were performed in triplicate for each group. One-way analysis of variance (ANOVA) was conducted using SPSS version 26.0 (IBM Corporation, Armonk, NY, USA). Bar charts, radar charts, and pie charts were created using Origin (version 2022). Principal component analysis (PCA) and partial least squares discriminant analysis (PLS-DA) were performed using Origin 2022 and Metaboanalyst 5.0 software. A visual heatmap for the content of flavor substances was generated using TBtools-II (version 2.323). Spearman correlation coefficients for key odor active compounds and key metabolites were calculated using CorHeatmap (https://www.genescloud.cn/chart/CorHeatmap, accessed on 15 January 2026), with significant correlations visualized in Cytoscape 3.10.1.

## 3. Results

### 3.1. Microbial-Growth-Dynamics Analysis

#### 3.1.1. Growth Kinetics and Acidification Characteristics of the Monoculture

**Figure 1:**
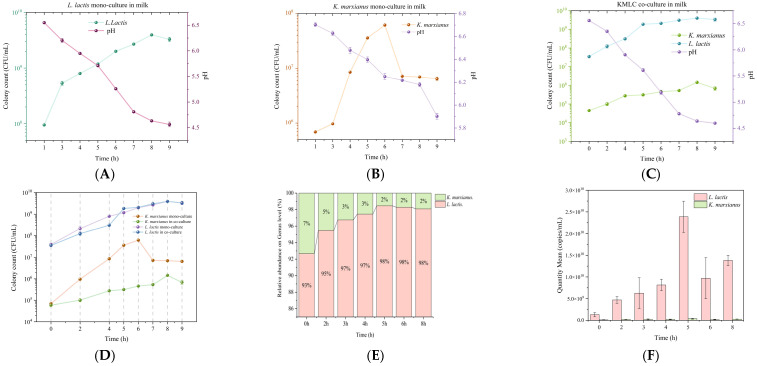
Microbial growth kinetics during the fermentation of milk: (**A**) changes in colony count and pH during fermentation in a *L. lactis* monoculture milk system; (**B**) changes in colony count and pH during fermentation in a *K. marxianus* monoculture milk system; (**C**) changes in colony count and pH during fermentation in a *K. marxianus* and *L. lactis* co-culture milk system; (**D**) colony counts changes at different fermentation time points in four groups of fermented milk; (**E**) changes in microbial absolute quantification at different fermentation time points in the KMLC group determined by 16S rRNA sequencing, ITS sequencing, and fluorescence quantification; (**F**) microbial composition changes at different fermentation time points in the KMLC group determined by 16S rRNA sequencing, ITS sequencing, and fluorescence quantification.

As shown in Figure 1A, the colony count of *L. lactis* during the fermentation process of fermented milk increased exponentially from 10^8^ CFU/mL to 3 × 10^9^ CFU/mL within the first 8 h, and then entered the stationary phase. In addition, pH decreased rapidly from an initial value of 6.5 to 4.5, and reached 4.6 at 8 h. The acidification rate peaked between 5 and 7 h. As shown in Figure 1B, the colony count of *K. marxianus* during the fermentation process of fermented milk increased exponentially from 10^5^ CFU/mL to 10^7^ CFU/mL between 2 and 6 h. However, the colony count rapidly decreased to 10^6^ CFU/mL between 6 and 7 h. After 7 h, the fermentation entered the stationary phase.

#### 3.1.2. Growth Kinetics and Acidification Characteristics of the Co-Culture

As shown in Figure 1C, in the co-culture system, both *K. marxianus* and *L. lactis* displayed an overall upward trend throughout the 0–9 h fermentation period. The growth rate of *K. marxianus* was similar to that of *L. lactis* during the 0–4 h. The growth rate of *L. lactis* rapidly increased after 4 h, reaching its peak colony counts at 5 h, and then gradually stabilized. The growth rate of *K. marxianus* gradually slowed down between 4 and 6 h, then rapidly increased, reaching its peak colony count at 8 h, followed by the decline phase. Figure 1C also illustrates the dynamic changes in pH during the co-culture process. The pH of the co-culture system decreased from 6.57 to 5.61 between 0 and 5 h. After 7 h, the acidification rate slowed, reaching 4.6 at 8 h.

#### 3.1.3. Synergistic Effects in the Co-Culture

As shown in Figure 1D, to compare the growth dynamics of *K. marxianus* and *L. lactis* in the co-culture system and the monoculture system, this study combined the growth curves of both microorganisms. During fermentation, the total colony count of *K. marxianus* in the co-culture system was 1 × 10^1^ to 1 × 10^3^ CFU/mL lower than that in the monoculture system. Compared to the monoculture system, the entry of *K. marxianus* into the logarithmic growth phase in the co-culture system was delayed by approximately 2 h, with the co-culture reaching this phase at 8 h and the monoculture at 6 h. Its growth accelerated significantly between 6 and 8 h, and it reached a peak at 8 h.

In the co-culture system, the growth trend of *L. lactis* during 0–4 h and 5–8 h was similar to that in the monoculture system. However, during the 4–5 h interval, the specific growth rate of *L. lactis* in the co-culture system (0.776 ± 0.0001 log CFU/mL/h) was significantly higher than that in the monoculture system (0.169 ± 0.011 log CFU/mL/h) (*p* < 0.01), representing a 4.6-fold increase. The growth curve of *L. lactis* in the co-culture system resembled that of the monoculture system. A transient increase of approximately 0.5 log CFU/mL was observed at 5 h, and both cultures reached similar stationary phase densities at 8 h.

In addition to the growth curves obtained by the plate pouring method, this study also analyzed the KMLC co-cultured fermented milk samples using 16S rRNA gene sequencing, ITS sequencing, and fluorescence quantification techniques (Figure 1E,F). Absolute copy numbers were quantified by fluorescence-based methods (Figure 1E). The percentage composition of the two strains in the KMLC co-culture system during fermentation was calculated from the absolute quantification data and was presented in Figure 1F. At the onset of fermentation (0 h), the microbial community was dominated by *L. lactis* (95% relative abundance), whereas *K. marxianus* represented a minor fraction (7% relative abundance). Subsequently, the relative abundance of *K. marxianus* declined to 2%, while that of *L. lactis* increased to 97%. After 4 h, the relative abundances of both species remained stable.

### 3.2. Sensory Evaluation

**Figure 2:**
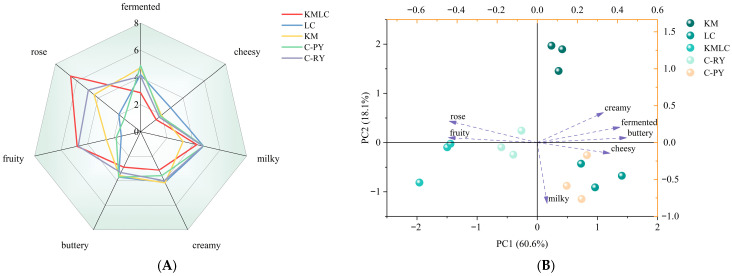

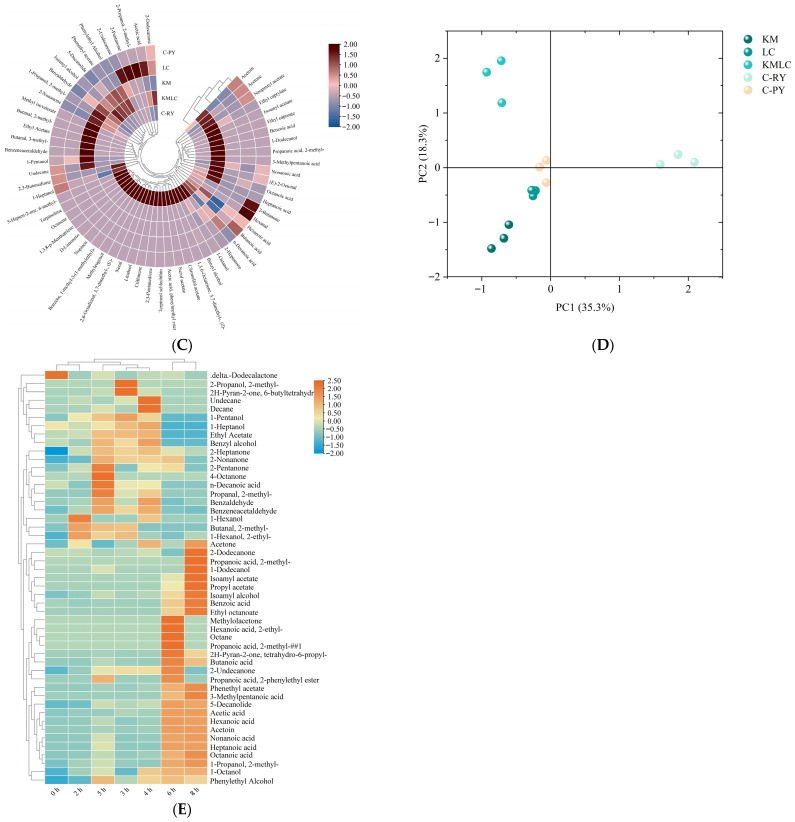
Sensory evaluation results and flavoromics analysis of different fermented milk: (**A**) sensory radar chart of five groups of fermented milk after 8 h of fermentation; (**B**) sensory PCA chart of five groups of fermented milk after 8 h of fermentation; (**C**) cluster heat map of flavor compounds in five types of fermented milk after 8 h of fermentation; (**D**) flavor compound PCA chart of five groups of fermented milk after 8 h of fermentation; (**E**) cluster heatmap of flavor compounds in KMLC group fermented milk during 0–8 h of fermentation. Notes: The KMLC group is a *K. marxianus* and *L. lactis* co-culture fermented milk system. The KM group is the *K. marxianus* monoculture fermented milk system. The LC group is the *L. lactis* monoculture fermented milk system. The C-PY group is commercial plain fermented milk, and the C-RY is commercial rose-aroma fermented milk. (**E**) Different colors represent different concentrations. (blue = low, yellow = moderate, orange = high).

Quantitative descriptive analysis (QDA) was employed to evaluate the sensory profiles of three experimental fermented milk samples (KM, LC, and KMLC) alongside two commercial fermented milk samples (C-PY and C-RY). These commercial fermented milks were selected based on sensory evaluation scores, as detailed in Figure S1. As shown in Figure 2A, the KMLC group exhibited the highest rose aroma intensity at 6.7 points, with fruit aroma intensity matching that of the C-RY group at 5 points. The milky, cream, and butter aromas were at average levels, while the fermented and cheese aromas were lower than those in the other samples. Currently, the sensory QDA in the main text focuses primarily on orthonasal evaluation (sniffing), whereas in-mouth (retronasal) tasting data are detailed in Figure S2.

Principal component analysis (PCA) was performed to visualize the sensory aroma attributes of fermented milk samples (Figure 2B). PCA was performed based on the intensities of seven aroma descriptors to distinguish samples with different starters. As shown in Figure 2B, PC1 and PC2 explained 60.6% and 18.1% of the total variance, respectively. The commercial plain fermented milk (C-PY) and the LC group were both concentrated in the fourth quadrant, primarily displaying milky and cheese aroma characteristics. Their overall flavor profiles were similar. The LC group exhibited a more pronounced cheese aroma, and its milky aroma intensity was slightly weaker compared to the C-PY group. The commercial rose-aroma fermented milk (C-RY) and the KMLC group were clustered in the third quadrant, primarily displaying floral and fruity flavor characteristics, which were negatively correlated with the fermented and cheese flavor aromas.

### 3.3. Flavoromics Analysis

#### 3.3.1. Flavoromics Analysis of Different Fermented Milk

VOCs in fermented milk were detected by HS-SPME-Arrow-GC-MS (Figure 2C). A total of 63 VOCs were identified in the five groups of fermented milk, including 12 alcohols, 7 aldehydes, 10 acids, 11 esters, 12 ketones, and 11 other compounds (Table S5). PCA was performed on the VOCs of the five groups of fermented milk, and the score plot is shown in Figure 2D. As shown in Figure 2D, the KMLC group exhibited significant differences in VOCs compared to the other four fermented milk samples (*p* < 0.05). Compared to the KMLC group, the concentrations of 23 and 27 VOCs were significantly reduced in the KM and LC groups, respectively. To evaluate the contribution of VOCs to the overall flavor, this study calculated the odor activity value (OAV) based on the ratio of their concentration to the odor threshold. As shown in Table S5, the KMLC group exhibited 15 VOCs with an OAV greater than one, whereas the KM and LC groups displayed 14 and 7 VOCs with an OAV greater than one, respectively.

#### 3.3.2. Dynamic Flavoromics Analysis During the Fermentation Process of KMLC Fermented Milk

**Figure 3:**
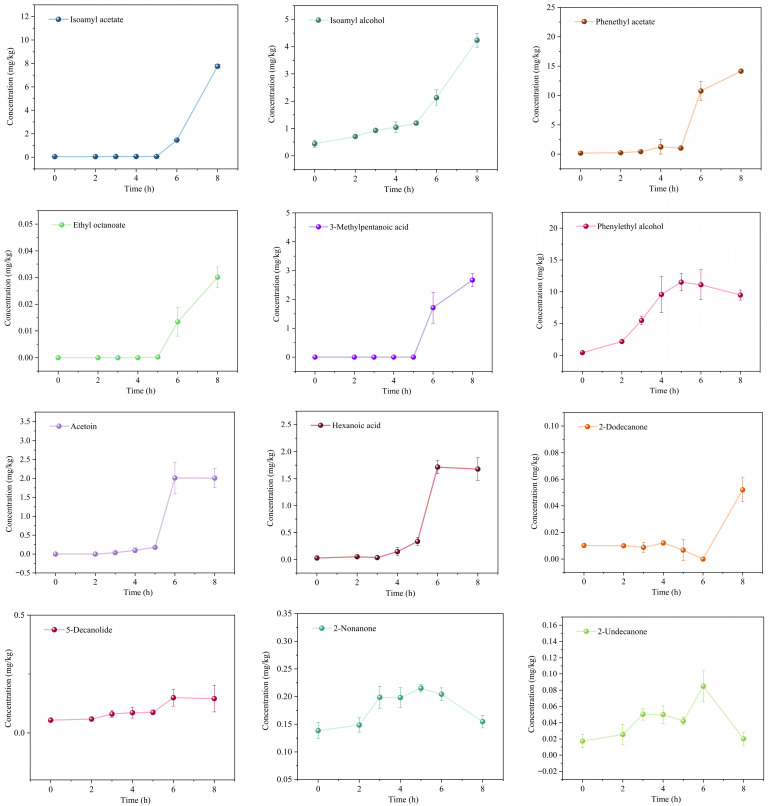

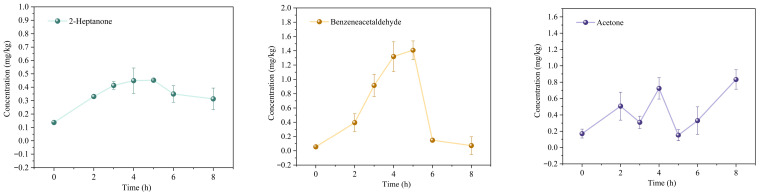
Changes in the concentration of the 15 key VOCs in the KMLC group fermented milk. Notes: volatile organic compounds (VOCs); the KMLC group is *K. marxianus* and *L. lactis* co-culture fermented milk system.

Dynamic changes in VOCs in KMLC fermented milk were analyzed. A total of 49 VOCs were identified by HS-SPME-Arrow-GC-MS, including 11 alcohols, four aldehydes, 11 acids, six esters, nine ketones, four heterocycles, and three other compounds (Table S5). The clustering heatmap visually presented the dynamic changes in VOCs in the fermented milk. To identify the key VOCs in the KMLC group fermented milk, this study further screened based on the OAVs at the fermentation endpoint (8 h). A total of 15 key VOCs were detected in the KMLC group fermented milk (Table 1), including two alcohols, one aldehyde, two acids, four esters, and six ketones (Figure 3).

Alcohols in KMLC fermented milk mainly included phenylethyl alcohol and isoamyl alcohol, and phenylethyl alcohol was the most abundant. Phenylethyl alcohol concentration increased gradually during the early stage (0–2 h), rose rapidly from 2 to 5 h, and then stabilized. Figure 3 shows the concentration changes of 15 key VOCs during the fermentation of KMLC group fermented milk. Among them, isoamyl acetate and phenethyl acetate had the highest concentrations. The concentrations of ten key VOCs, including isoamyl acetate, phenethyl acetate, 5-decanolide, ethyl octanoate, isoamyl alcohol, phenylethyl alcohol, acetoin, 2-dodecanone, 3-methylpentanoic acid, and hexanoic acid, continuously increased and eventually stabilized. In contrast, the concentrations of five VOCs, including 2-nonanone, 2-undecanone, 2-heptanone, acetone, and benzeneacetaldehyde, fluctuated significantly during fermentation and showed a decreasing trend. Moreover, changes in isoamyl alcohol and phenylethyl alcohol concentrations were consistent with the growth kinetics of *K. marxianus* in co-culture.

### 3.4. Metabolomics Analysis

#### 3.4.1. Metabolomics Analysis of KMLC Group Fermented Milk

**Figure 4:**
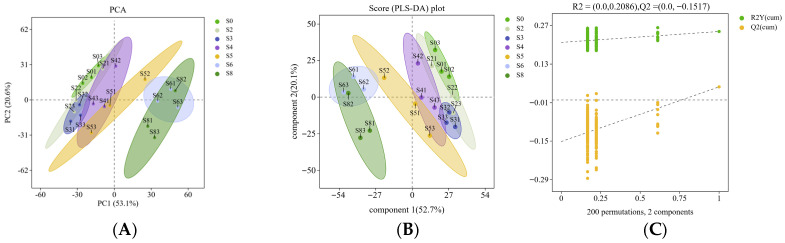

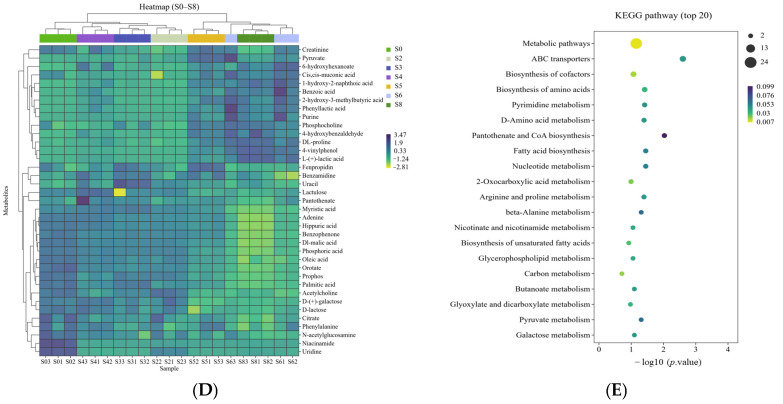
Metabolomics analysis of KMLC group fermented milk: (**A**) PCA; (**B**) PLS-DA; (**C**) PLS-DA permutation test plot; (**D**) differential metabolite clustering heat map analysis; (**E**) Kyoto encyclopedia of genes and genomes (KEGG) pathway analysis of key metabolites. Notes: S0–S8 represented the samples from 0 h to 8 h in the fermentation process of KMLC fermented milk, respectively. (**E**) The horizontal coordinates indicate the negative logarithmic transformation of the *p*-value, and the vertical coordinates indicate the pathway name. The size of the circle indicates the count, i.e., the number of metabolites annotated to the pathway; the color of the circle corresponds to the corrected *p*-value, with red to blue being the more significant. Metabolites mapped to metabolic pathways were primarily classified under metabolism.

In this study, liquid chromatography–tandem mass spectrometry (LC-MS/MS) was used for untargeted metabolomics analysis of non-volatile metabolites in the KMLC fermented milk samples, resulting in the detection of 1741 metabolites. PCA was performed on the metabolites in fermented milk at different fermentation stages (Figure 4A). The PC1 and PC2 accounted for 53.10% and 20.60% of the variance, respectively, with a cumulative variance contribution of 73.70%. As shown in Figure 4A, there were significant differences in the metabolites of fermented milk between the early and late stages of fermentation. PLS-DA was performed to analyze samples at different fermentation stages (Figure 4B). The PLS-DA model was validated by 200 permutation tests with no overfitting (Figure 4C).

#### 3.4.2. Key Metabolites Analysis of KMLC Group Fermented Milk

Differential metabolites were selected using variable importance in projection (VIP) > 1 and *p* < 0.05 as the criteria. As shown in Table S6, a total of 37 compounds were selected, including eight organic acids and derivatives, six lipids and lipid-like molecules, six aromatic compounds, five organoheterocyclic compounds, five organic oxygen compounds, two organic nitrogen compounds, one nucleoside, nucleotide, and analogue, and four other metabolites. These substances were key metabolites in the fermentation process. The clustering heatmap revealed the dynamic changes in key metabolites during the fermentation process (Figure 4D). As the fermentation proceeded, concentrations of 17 metabolites decreased gradually, including lactic acid, lactose, galactose, palmitic acid, hippuric acid, and orotate. In contrast, concentrations of 12 metabolites increased gradually with fermentation time, including benzoic acid and phenylacetic acid.

The organic acids detected in the KMLC group fermented milk mainly included malic acid, citric acid, lactic acid, orotate, benzoic acid, and phenylacetic acid. During fermentation, the concentrations of malic acid, citric acid, lactic acid, and orotate decreased, while the concentrations of phenylacetic acid and benzoic acid increased. The organic oxygen compounds detected in the KMLC group fermented milk mainly included lactose, galactose, and N-acetylglucosamine (GlcNAc). The lipid compounds detected in the KMLC group fermented milk included myristic acid, oleic acid, and palmitic acid. The amino acid compounds detected in the KMLC group fermented milk mainly included hippuric acid and phenylalanine.

Kyoto encyclopedia of genes and genomes (KEGG) database matching and metabolic pathway enrichment analysis of 37 key metabolites identified a total of 44 metabolic pathways. Figure 4E presented the top 20 pathways ranked by significance during fermentation, with 13 of these pathways having a false discovery rate (FDR) less than 0.05. These pathways included eight related to amino acid metabolism, 8 global and overview pathways, 12 related to carbohydrate metabolism, five related to lipid metabolism, four related to cofactor and vitamin metabolism, two related to nucleotide metabolism, four related to other amino acid metabolism, and one related to energy metabolism. Among these, citric acid metabolism and amino acid metabolism were the most dominant pathways.

### 3.5. Correlation Network Analysis

**Figure 5:**
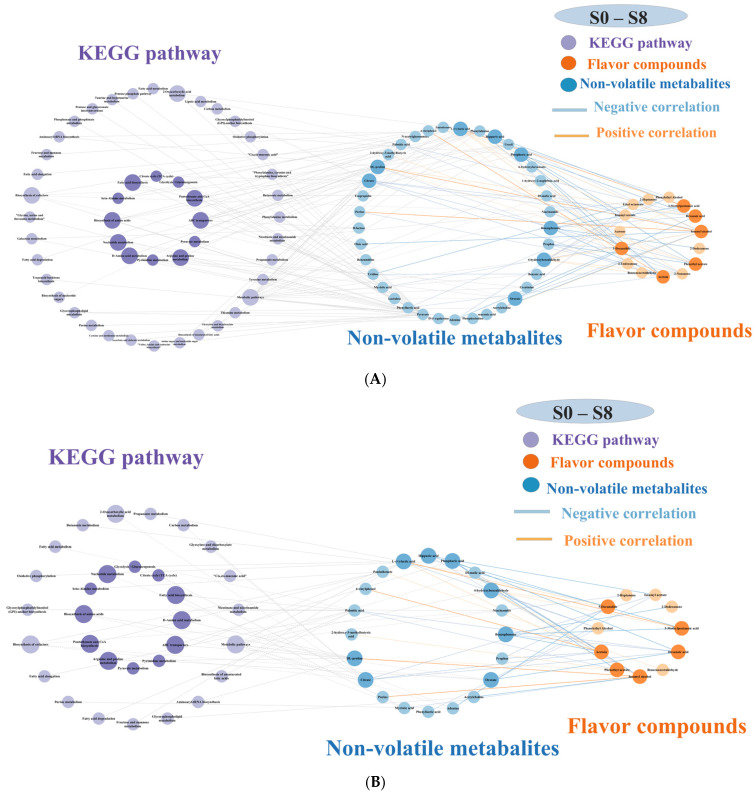

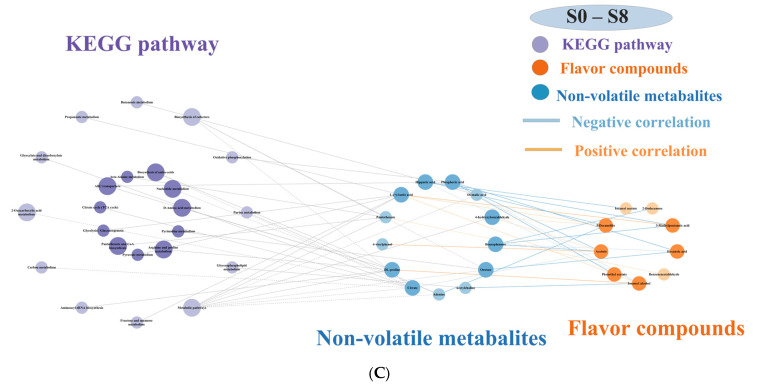
Network diagram of metabolic pathways, key metabolites, and key volatile organic compounds (VOCs): (**A**) correlations of metabolic pathways, key metabolites, and key VOCs (|r| > 0.7, *p* < 0.05); (**B**) correlations of metabolic pathways, key metabolites, and key VOCs (|r| > 0.8, *p* < 0.05); (**C**) correlations of metabolic pathways, key metabolites, and key VOCs (|r| > 0.9, *p* < 0.05). Notes: the size of the circles represents the number of associated variables; larger circles indicate more associated variables. Darker purple circles indicate *p* < 0.05, while lighter purple circles indicate *p* ≥ 0.05. Solid lines between purple and blue circles indicate upregulation, dashed lines indicate downregulation. The darker the color of the blue and yellow circles, the greater the difference. Two circles connected by pink lines are positively correlated, while those connected by blue lines are negatively correlated (the thickness of the lines represents the strength of the correlation; thicker lines indicate stronger correlations).

The results of Spearman correlation analysis indicated that the top ten VOCs with the most significant OAV contributions and thirteen key metabolites (|r| > 0.9, *p* < 0.05) were selected for visual correlation network analysis (Figure 5). As shown in Figure 5, eight key VOCs, isoamyl acetate, isoamyl alcohol, 3-methylpentanoic acid, hexanoic acid, phenylethyl acetate, acetoin, 2-dodecanone, and 5-decanolide, were significantly negatively correlated with eight key metabolites, including hippuric acid, citric acid, adenine, orotate, DL-malic acid, benzophenone, lactic acid, and phosphoric acid. Key metabolites were mapped onto metabolic pathways to screen for critical routes (*p* < 0.05).

## 4. Discussion

This study employed a multi-omics approach combining flavoromics and metabolomics analysis to explore the biosynthesis mechanisms of the characteristic rose aroma of the fermented milk produced by a co-culture of *K. marxianus* and *L. lactis*. The acidification rate peaked between 5 and 7 h, which may be associated with lactic acid production during lactose metabolism by *L. lactis* in monoculture. The decline in *K. marxianus* colony count between 6 and 7 h may be attributed to the strain entering the death phase, as the depletion of nutrients in the fermented milk and the production of secondary metabolites such as alcohol inhibited the growth of *K. marxianus*. In addition, the pH decreased from 6.5 to 5.9 in the monoculture system, indicating weak acidification capacity by *K. marxianus*. The slow pH change indicated that yeast metabolism is primarily respiratory, with lactic acid production not being the primary metabolic pathway. The delayed acidification after 7 h in the co-culture system might be associated with the buffering effect of increased *K. marxianus* metabolic activity. Specifically, the rapid acid production by *L. lactis* within 0-7 h created a low-pH stress environment (pH < 6.00) that likely inhibited *K. marxianus* growth during the 7–8 h interval. Subsequently, the slowing of acidification may reflect enhanced yeast metabolic activity, potentially through the consumption of *L. lactis* or the production of alkaline metabolites such as ammonia.

The distinct growth patterns of the two strains in co-culture could be explained by the early-stage acid stress. The rapid acid production by *L. lactis* at 0–6 h lowered the pH and inhibited the proliferation of *K. marxianus*. After 6 h, *L. lactis* entered the stationary phase with reduced acid production, allowing *K. marxianus* to adapt to the acidic environment and grow rapidly from 6 to 8 h, reaching the maximum count at 8 h. However, restricted by nutrient availability and the competitive advantage of *L. lactis*, the final biomass of *K. marxianus* in co-culture was still lower than that in monoculture. Nutrient competition existed between *K. marxianus* and *L. lactis* in the milk matrix. Notably, the specific growth rate of *L. lactis* at 4–5 h in co-culture was 4.6 times higher than that in monoculture, suggesting a growth-promoting effect of *K. marxianus* on *L. lactis*. This synergistic interaction might be attributed to the secondary metabolites (e.g., CO_2_, vitamins, ethanol) produced by *K. marxianus*, which provided additional nutrients for *L. lactis* and accelerated its proliferation [29]. This result was consistent with previous reports that *K. marxianus* could promote the growth and fermentation efficiency of lactic acid bacteria [30].

The 16S rRNA gene amplicon sequencing was performed for the LAB and the ITS amplicon sequencing for yeast to verify strain purity, confirm taxonomic identity, and exclude possible contaminants. Because these two amplicon libraries could not be combined into a single quantitative run, qPCR was employed to determine the absolute copy numbers of bacteria and fungi per gram of fermented milk, with relative abundance calculated from absolute quantification data. The results further confirmed the microbial community composition, species relative abundance, and their proportions in the co-culture system, which were consistent with the findings obtained from the plate count method.

The sensory profiles of fermented milk samples were assessed using QDA. The orthogonal experiments and sensory results in Tables S1–S3 confirmed that the sensory scores of the KMLC group were improved by the addition of exogenous phenylalanine. The main reason was that exogenous phenylalanine was primarily catabolized via the Ehrlich pathway, involving transamination to phenylpyruvate, subsequent decarboxylation to phenylacetaldehyde, and finally reduced to 2-phenylethanol, which was identified as the key contributor to rose aroma [31]. Principal component analysis (PCA), as a dimensionality reduction technique, could be used to visualize the sensory aroma attributes of fermented milk samples [32]. PC1 and PC2 explained 60.6% and 18.1% of the total variance, indicating that the constructed model effectively reflected the fundamental information of the samples, respectively. The commercial plain fermented milk (C-PY) and the LC group were concentrated in the fourth quadrant, primarily displaying milky and cheese aroma characteristics. Although their overall flavor profiles were similar, the LC group exhibited a more pronounced cheese aroma with slightly weaker milky intensity compared to the C-PY group. In contrast, the commercial rose-aroma fermented milk (C-RY) and the KMLC group clustered in the third quadrant, primarily displaying floral and fruity flavor characteristics that were negatively correlated with fermented and cheese aromas. The distinct quadrant distribution of the KMLC, LC, and KM groups indicates that co-cultivation of *K. marxianus* and *L. lactis* produced fermented milk with more pronounced floral and fruity aromas compared to mono-strain fermentation, consistent with previous findings that yeasts produce more alcohols, acids, esters, and other flavor compounds during fermentation, thereby enhancing sensory scores [33]. This suggested that the differences in sensory evaluation were closely related to microbial metabolism and the generation of VOCs.

VOCs are key quality attributes of fermented milk [34]. It is generally considered that compounds with an OAV ≥ 1 make a significant contribution to the overall flavor [35]. Significant differences were observed in VOC profiles and OAV characteristics between the KMLC fermented milk and the other four groups. This may account for the flavor differences between co-cultured and monocultured fermented milk. Sensory evaluation and VOCs analysis confirmed that the co-culture system produced fermented milk with a more prominent floral and fruity aroma compared to the monoculture system. Therefore, to explore the sources of the more prominent floral and fruity aromas in the co-culture system, the flavor and metabolic changes in the KMLC group fermented milk during the fermentation process were further analyzed in this study.

15 key VOCs were screened in the KMLC group based on OAV, including esters, alcohols, ketones, acids, and aldehydes. Esters such as isoamyl acetate and phenethyl acetate were the most abundant, providing strong sweet, fruity, and rose-like aromas, and alleviating the pungent taste of organic acids [36]. Ketones (acetoin, 2-nonanone, etc.) were mainly derived from fatty acid β-oxidation and amino acid metabolism [37], imparting creamy, fruity, and floral notes [38,39]. Acetoin significantly enhanced the creamy mouthfeel of fermented milk [40]. Phenylethyl alcohol, the core substance of rose aroma [41], was mainly synthesized by *K. marxianus* via the Ehrlich pathway using exogenous phenylalanine as the precursor [31], and its dynamic change was consistent with the growth trend of *K. marxianus*. Organic acids such as 3-methylpentanoic acid and hexanoic acid were produced by *L. lactis* through fat hydrolysis and oxidation, enhancing flavor complexity without causing excessive sourness [42,43]. Benzeneacetaldehyde contributed sweet honey and floral notes, further enriching the characteristic aroma [44]. Moreover, the concentration changes in isoamyl alcohol and phenylethyl alcohol were consistent with the growth kinetics of *K. marxianus* in the co-culture system, suggesting that these compounds were likely produced by *K. marxianus*.

Untargeted metabolomics analysis served as an effective tool for identifying metabolites such as amino acids, lipids, and organic acids formed under different conditions [39,45]. In this study, LC-MS/MS detected 1741 metabolites. PCA revealed significant differences in metabolite profiles between early and late fermentation stages, while PLS-DA confirmed that fermentation time significantly impacted metabolite production. After 200 permutation tests, the PLS-DA model showed no overfitting, indicating accurate and effective metabolite selection between different groups [46]. Notably, consistent clustering patterns were observed in both metabolomic and flavoromic analyses (Figure 2E and Figure 4A,B,D). Samples collected between 0 and 4 h were clustered as a distinct group, whereas those from 5 to 8 h were grouped into a separate cluster. This temporal partitioning indicated that a metabolic shift occurred at approximately 4–5 h of fermentation, likely corresponding to the transition from the early acidification phase (dominated by *L. lactis* metabolism) to the late aroma formation phase (driven primarily by *K. marxianus* activity). The consistency of results across different analytical platforms further confirmed this metabolic transition.

Differential metabolites were selected using variable importance in projection (VIP) > 1 and *p* < 0.05 as the criteria [47]. The decrease in the concentrations of malic acid and citric acid may have resulted from the metabolic utilization by the fermentation agents [48]. Malic acid was generated through the coordinated action of yeast cytoplasmic malate dehydrogenase (MDH) and nicotinamide adenine dinucleotide phosphate (NADP +), and it could also be converted into lactic acid by LAB [49]. Citric acid was metabolized by LAB into lactate, diacetyl, and other compounds, further influencing the flavor [50]. Benzoic acid was an antimicrobial compound, typically synthesized by LAB during fermentation. It could also be produced through the conversion of hippuric acid or the breakdown of phenylalanine, helping to maintain the quality and stability of fermented milk during storage [51]. Phenylacetic acid was a naturally occurring antimicrobial compound that was produced by LAB. Its excessive accumulation could inhibit the growth and acid production capacity of the LAB [52,53]. Orotate, as an intermediate in nucleic acid synthesis, has its concentration changes were co-regulated by the initial concentration of the raw milk and the microbial activity during fermentation [54].

The decrease in the concentrations of lactose and galactose may have been due to the utilization of lactose by LAB to produce lactic acid, while *K. marxianus* could also metabolize lactose and galactose to generate pyruvate and 2-phenylethanol. GlcNAc primarily originated from the oligosaccharide chains of glycoproteins and glycolipids in the raw milk. During the early stages of fermentation, the proteases and glycosidases secreted by *L. lactis* and *K. marxianus* gradually hydrolyzed macromolecules, releasing free GlcNAc and other monosaccharides or amino sugars [55], leading to a temporary increase or maintenance of GlcNAc concentrations in the early fermentation stages. As fermentation progressed, GlcNAc could have been utilized by microorganisms as a carbon and nitrogen source [56], leading to a decreasing trend in its concentration.

The decrease in lipid content during the fermentation process was likely due to the depletion of carbon sources such as lactose in the later stages of fermentation, with lipids becoming the primary energy source for the microorganisms. Under stress conditions such as low pH and nutrient competition, microorganisms may undergo lipid catabolism. This was likely because *K. marxianus* and *L. lactis* gradually broke down fatty acids through β-oxidation, providing energy for the cells and supplying structural units for the synthesis of cell membrane phospholipids [57]. Additionally, fatty acids can serve as precursor molecules for the synthesis of various volatile flavor compounds, such as phenylacetaldehyde and ethyl hexanoate [58,59].

Spearman correlation analysis was used to examine the relationship between VOCs and non-volatile metabolites, and the significantly correlated metabolite pairs were mapped to metabolic pathways to construct a visual correlation network. Among them, positively correlated metabolites are mainly co-generated with VOCs, while negatively correlated metabolites may serve as precursors for flavor formation. Citric acid was cleaved via the down-regulated energy-metabolism pathway of *L. lactis* to yield oxaloacetate and acetyl-CoA. Acetyl-CoA was subsequently channeled into the diacetyl/acetoin pathway and converted to acetoin, which confers a creamy aroma, whereas oxaloacetate sustained tricarboxylic-acid-cycle homeostasis [60]. Adenine, a core nucleotide component, was degraded to glyoxylate and glycerate, which fed the central carbon metabolism and supplied carbon skeletons for key VOCs such as 5-decanolide [61]. DL-malic acid was transformed into pyruvate through the butanoate pathway, and pyruvate then served as a precursor for 1-butanol and isoamyl alcohol via the Ehrlich pathway [62]. Lactic acid was converted to pyruvate by glycolysis, pyruvate entered the shikimate pathway for phenylalanine biosynthesis, and phenylalanine was further deaminated, decarboxylated, and reduced to phenylethyl alcohol [63]. Phosphoric acid was processed through the ABC transporter system to generate acyl-CoA, acetyl-CoA, and acetoin, thereby promoting the formation of hexanoic acid and phenethyl acetate [64]. Orotate supplied the tricarboxylic acid cycle with intermediates such as succinate, which additionally contributed to flavor development [65]. Collectively, *K. marxianus* and *L. lactis* coordinately utilized the citrate, nucleotide, pyruvate, and phosphate metabolic pathways, thereby generating the characteristic floral and fruity aroma of the fermented milk.

## 5. Conclusions

This study employed a multi-omics approach combining flavoromics and metabolomics analysis to explore the biosynthesis mechanisms of the characteristic rose aroma of the fermented milk produced by a co-culture of *K. marxianus* and *L. lactis*. The orthogonal experiment confirmed that exogenous phenylalanine supplementation significantly enhanced the sensory scores and rose aroma intensity of the fermented milk compared to the non-supplemented group, indicating that phenylalanine serves as an effective precursor for aroma enhancement. Therefore, a detailed analysis of the flavor and metabolomic profiles was performed on the phenylalanine-supplemented group to elucidate the metabolic mechanisms. The microbial growth kinetics indicated that, under the co-culture system, *K. marxianus* could promote the growth of *L. lactis* and synergistic effects in the co-culture. The sensory profiles and VOCs of the KMLC group showed significant differences (*p* < 0.05) compared to the KM, LC, and the commercial fermented milk group, with the KMLC group exhibiting a more pronounced rose-like aroma. During the 8 h fermentation, 15 key VOCs were identified in the KMLC group based on their OAVs, and 37 key metabolites were identified with VIP > 1 and *p* < 0.05. The key metabolites were enriched in 44 metabolic pathways, with citrate metabolism and amino acid metabolism being the dominant pathways. Spearman correlation analysis showed a significant correlation between key metabolites and key VOCs. These key metabolites play a crucial role in the formation of floral and fruity aromas by participating in metabolic pathways such as citric acid metabolism, nucleotide metabolism, and phosphate metabolism. In conclusion, this study demonstrated that *K. marxianus* and *L. lactis*, as a composite starter culture, can render and enhance the unique rose aroma of fermented milk, underscoring the significant yet unexploited potential of *K. marxianus*-composite microbial consortia in driving flavor innovation for the fermented dairy industry. Future research will focus on identifying regulatory targets of the metabolic pathways for steady and optimal production of the rose aroma compounds to accommodate the industrial-scale continuous fermentation, thereby underpinning the high-quality development as well as flavor innovation of the dairy industry.

## Figures and Tables

**Table 1:** The concentrations and descriptions of the 15 key VOCs identified in the KMLC fermented milk.

Categorye	Flavor Compounds	CAS	OAV	Concentration (mg/kg)	Odor Description
Esters	Isoamyl acetate	123-92-2	51,765.14	7.76 ± 0.15	Sweet, fruity, banana
	Phenethyl acetate	103-45-7	56.70	14.15 ± 0.18	Rose, honey, floral, fruity
	5-Decanolide	705-86-2	2.19	0.14 ± 0.07	Peach, fruity, creamy
	Ethyl octanoate	106-32-1	1.58	0.03 ± 0.00	Pineapple, creamy, waxy, fruity, winey
Alcohols	Isoamyl alcohol	123-51-3	1057.99	4.23 ± 0.24	Alcoholic, whiskey, fruity, banana
	Phenylethyl alcohol	60-12-8	16.81	9.48 ± 0.79	Rose
Ketones	Acetoin	513-86-0	143.50	2.01 ± 0.23	Milk, buttery, creamy
	2-Nonanone	821-55-6	3.77	0.15 ± 0.01	Sweet, green
	2-Undecanone	112-12-9	3.72	0.02 ± 0.01	Fruity, creamy, fatty, orris, floral
	2-Heptanone	110-43-0	2.24	0.31 ± 0.08	Sweet, herbal, coconut, woody, fruity
	2-Dodecanone	6175-49-1	1.25	0.05 ± 0.01	Fruity, citrus, floral, orange
	Acetone	67-64-1	1.00	0.83 ± 0.11	Apple, pear
Aldehydes	Benzeneacetaldehyde	122-78-1	11.47	0.07 ± 0.13	Green, sweet, floral, hyacinth, honey, cocoa
Acids	3-Methylpentanoic acid	105-43-1	58.23	2.68 ± 0.22	Green, fruity, sweaty, cheesy
	Hexanoic acid	142-62-1	1.88	1.67 ± 0.21	Cheesy, fatty, sweaty

Notes: CAS registry number (CAS); odor activity value (OAV); volatile organic compounds (VOCs). The KMLC group is the *K. marxianus* and *L. lactis* co-culture fermented milk system (KMLC). All odor descriptions are sourced from https://www.thegoodscentscompany.com/categories.html, accessed on 10 December 2025.

## Data Availability

The original contributions presented in this study are included in the article/Supplementary Materials. Further inquiries can be directed to the corresponding authors.

## Supplementary Materials

The following supporting information can be downloaded at: https://www.mdpi.com/article/10.3390/metabo16040235/s1. Figure S1: Sensory of different commercial fermented milk. Figure S2. Sensory radar chart of five groups of fermented milk after 8 h of fermentation (in-mouth tasting data). Table S1: Single-factor experimental optimization of fermentation parameters and results. Table S2: Factors and levels of orthogonal test. Table S3: Results and analysis of orthogonal experiment. Table S4: List of sensory evaluation attributes for fermented milk. Table S5: List of VOCs identified in five groups of fermented milk. Table S6: List of 37 key metabolites identified in KMLC fermented milk.
