# Supplementary material for: Synergistic Growth and Metabolic Interactions of Kluyveromyces marxianus and Lactococcus lactis in Rose-Aroma Fermented Milk Revealed by Integrated Flavoromics and Metabolomics
Source: Metabolites. 2026 Mar 31;16(4):235. doi: 10.3390/metabo16040235 (PMC13118117; doi:10.3390/metabo16040235)
Supplement: Supplementary file 1 [file metabolites-16-00235-s001.zip › metabolites-4180957-supplementary.pdf]

Supplementary Materials

Synergistic growth and metabolic interactions of *Kluyveromyces marxianus* and *Lactococcus lactis* in rose-aroma fermented milk revealed by integrated flavoromics and metabolomics

Jiawen Liu[1,†], Ziyan Yue[1,†], Yuyao He[1], Xinchi Jiang[1], Hong Zeng[1,2,*], Yanbo Wang[1,2,*]

[1] School of Food and Health, Beijing Technology and Business University, Beijing, 100048, P. R. China

[2] Key Laboratory of Geriatric Nutrition and Health (Beijing Technology and Business University), Ministry of Education, Beijing, 100048, P. R. China

* Correspondence: zenghong@btbu.edu.cn (H.Z.); wyb1225@163.com (Y.W)

† These authors contributed equally to this work.

2. Materials and methods

2.2 Preparation of rose-aroma fermented milk samples

Table S1

Single-factor experimental optimization of fermentation parameters

Parameters	Level	Sensory Score	Optimal Parameter
Fermentation temperature	28°C	61	
	32°C	73	
	36°C	87	√
	40°C	80	
	44°C	72	
Phenylalanine supplementation	0	70	
	0.20%	90	√
	0.40%	80	
	0.60%	86	
	0.80%	78	
Inoculum size	1%	85	
	2%	77	
	3%	78	
	4%	87	√
	5%	83	

Table S2

Parameters and levels of the orthogonal experiment

Level	A<br>Fermentation temperature<br>(°C)	B<br>Phenylalanine supplementation<br>(%)	C<br>Inoculum size<br>(%)
1	32	0.1	3
2	36	0.2	4
3	40	0.4	5

Table S3

Results of the orthogonal experiment

Run	A<br>Fermentation temperature	B<br>Phenylalanine supplementation	C<br>Inoculum size	Empty	Sensory score
1	1	1	1	1	76.5
2	1	2	2	2	85.0
3	1	3	3	3	72.5
4	2	1	2	3	84.0
5	2	2	3	1	87.0
6	2	3	1	2	81.0
7	3	1	3	2	71.0
8	3	2	1	3	83.0
9	3	3	2	1	79.0
K1	78.0	77.2	80.2	80.8	
K2	84.0	85.0	82.7	79.0	
K3	77.7	77.5	76.8	79.8	
Range (R)	6.3	7.8	5.9	1.8	
Order of importance			B＞A＞C		
Optimal level			A2B2C2		

Based on the results of single-factor and orthogonal experiments, the optimal parameters for rose-aroma fermented milk were determined as a fermentation temperature of 36°C, an inoculum size of 4%, and a phenylalanine supplementation of 0.2%.

2.4 Sensory evaluation

Table S4

List of sensory evaluation attributes for fermented milk.

Descriptor	Definition	Reference substance
Fermented	The characteristic aroma produced by the fermentation of dairy products	Bajifu Small Triangle Cheese
Cheesy	The taste of fresh cheese with a characteristic sour odor	Cheddar or mozzarella
Milky	Characteristic odors associated with pure milk	Pure milk
Creamy	Oil flavor from fresh cream or sweet cream	Nestle light cream
Buttery	Fresh butter gives off a creamy, fresher-than-dairy flavor	President light butter cubes
Fruity	A rich aroma of ripe fruits and a blend of sweet and floral notes	Fruity fermented milk mix
rose	A rich aroma of rose	Phenylethyl alcohol (2.89 mg/L aqueous solution)

3 Results and discussion

3.2 Sensory analysis

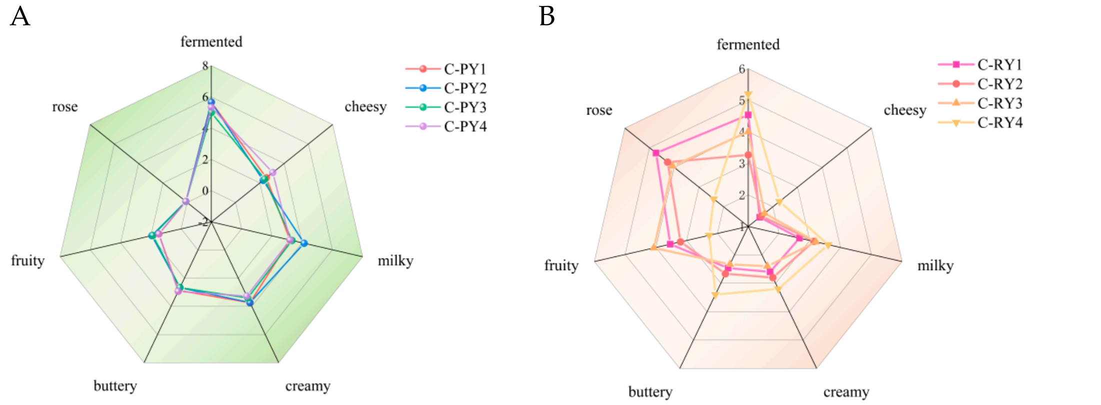

Figure S1. Sensory of different commercial fermented milk: (A) Sensory radar chart of four commercial plain fermented milk; (B) Sensory radar chart of four commercial rose-aroma fermented milk.

Notes: The C-PY group is commercial plain fermented milk, and C-RY is commercial rose-aroma fermented milk. A: The C-PY1 group is Jian'ai sucrose-free plain fermented milk, C-PY2 group is Hema 0 sucrose plain fermented milk, C-PY3 group is Yiming 0 sucrose plain fermented milk and C-PY4 group is Horun 0 sucrose plain fermented milk. B: The C-RY1 group is Dali Ranch fermented milk, C-RY2 group is Dali typical fermented milk, C-RY3 group is Yang Da fermented milk and C-RY4 group is New Hope Xuelan fermented milk.

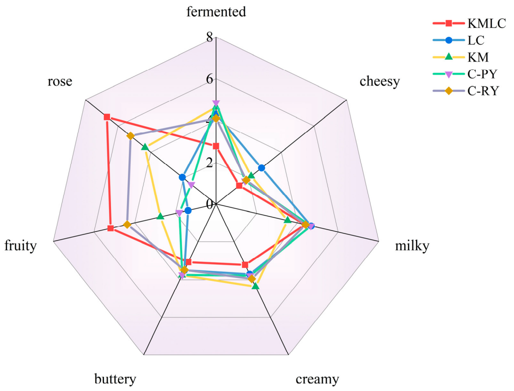

Figure S2. Sensory radar chart of five groups of fermented milk after 8 h of fermentation (in-mouth tasting data).

Notes: The KMLC group is *K. marxianus* and *L. lactis* co-culture fermented milk system. The KM group is *K. marxianus* mono-culture fermented milk system; The LC group is *L. lactis* mono-culture fermented milk system; The C-PY group is commercial plain fermented milk, and C-RY is commercial rose-aroma fermented milk.

3 Results and discussion

Table S5

List of VOCs identified in five groups of fermented milk.

Category	Flavor compounds	CAS	Concentration（mg/kg）				
			KMLC	LC	KM	C-PY	C-RY
Ketones	Acetone	67-64-1	0.83±0.11[a]	0.2±0.07[b]	0.29±0.33[ab]	0.57±0.43[ab]	0.19±0.02[b]
	6-Methyl-5-heptene-2-one	110-93-0	ND	ND	ND	ND	0.01±0.00[a]
	2-Undecanone	112-12-9	0.02±0.01[ab]	0.08±0.09[a]	0.06±0.01[a]	0.01±0.00[b]	0.02±0.00[ab]
	3-Heptanone, 2-methyl-	13019-20-0	1.0±0.00[a]	1.0±0.00[a]	1.0±0.00[a]	1.0±0.00[a]	1.0±0.00[a]
	2-Heptanone	110-43-0	0.31±0.08[bc]	0.27±0.04[c]	0.46±0.06[b]	0.25±0.20[c]	1.3±0.90[a]
	Acetoin	513-86-0	2.01±0.23[a]	0.04±0.02[c]	ND	1.39±0.48[b]	0.84±0.05[b]
	2,3-Pentanedione	600-14-6	ND	ND	ND	ND	0.02±0.00[a]
	2-Nonanone	821-55-6	0.15±0.01[a]	0.07±0.11[ab]	0.24±0.05[a]	0.06±0.05[b]	0.11±0.01[ab]
	2-Dodecanone	6175-49-1	0.05±0.01[a]	0.05±0.07[a]	ND	0.03±0.05[a]	ND
	2-Pentanone	107-87-9	ND	0.07±0.03[a]	ND	ND	ND
	2-Butanone	78-93-3	ND	ND	ND	0.13±0.03[a]	0.05±0.05[b]
	2,3-Butanedione	431-03-8	ND	ND	ND	0.35±0.37[b]	0.67±0.08[a]
Alcoholse	2-Methyl-1-propanol	78-83-1	0.08±0.02[a]	ND	0.17±0.06[a]	ND	ND
	1-Heptanol	111-70-6	ND	ND	0.00±0.01[a]	0.01±0.01[a]	0.01±0.02[a]
	3-Methylbutanal	590-86-3	ND	ND	0.13±0.03[a]	ND	ND
	Isoamyl alcohol	123-51-3	4.23±0.24[a]	ND	2.39±0.35[b]	ND	ND
	1-Octanol	111-87-5	ND	ND	ND	ND	0.02±0.01[a]
	tert-Butanol	75-65-0	ND	ND	ND	ND	ND
	1-Pentanol	71-41-0	ND	0.01±0.00[b]	0.03±0.01[a]	0.00±0.10[ab]	ND
	1-Dodecanol	112-53-8	0.01±0.01[a]	ND	ND	ND	ND
	Benzyl alcohol	100-51-6	ND	0.01±0.00[c]	ND	ND	0.16±0.14[a]
	Phenylethyl alcohol	60-12-8	9.48±0.79[a]	0.10±0.02[d]	7.95±4.48[a]	0.01±0.01[d]	1.32±0.25[b]
	Linalool	78-70-6	ND	ND	ND	ND	0.36±0.32[a]
	Alpha-terpineol	98-55-5	ND	ND	ND	ND	0.08±0.01[a]
Acidse	Acetic acid	64-19-7	1.21±0.36[b]	1.83±0.36[a]	0.15±0.15[c]	0.49±0.47[c]	0.14±0.03[c]

	Hexanoic acid	142-62-1	1.67±0.21[a]	1.02±0.28[b]	0.13±0.04[c]	1.17±0.12[b]	1.39±0.34[ab]
	Heptanoic acid	111-14-8	0.03±0.02[a]	ND	ND	0.0±0.01[a]	0.02±0.01[a]
	Octanoic acid	124-07-2	1.86±0.11[a]	0.48±0.12[b]	0.20±0.10[b]	0.22±0.09[b]	0.52±0.21[b]
	Nonanoic acid	112-05-0	0.16±0.11[a]	0.01±0.00[b]	0.05±0.03[ab]	0.05±0.04[ab]	0.01±0.00[b]
	Isobutyric acid	79-31-2	4.07±1.93[a]	0.02±0.03[b]	0.01±0.01[b]	ND	ND
	Decanoic acid	334-48-5	ND	0.06±0.05[a]	0.03±0.04[a]	0.01±0.01[a]	0.06±0.03[a]
	Benzoic acid	65-85-0	0.25±0.28[a]	ND	ND	ND	ND
	Butyric acid	107-92-6	0.12±0.20[a]	0.08±0.03[a]	ND	0.21±0.02[a]	0.21±0.05[a]
	3-Methylpentanoic acid	105-43-1	2.68±0.22[a]	0.01±0.01[b]	ND	ND	ND
Esters	Ethyl acetate	141-78-6	ND	ND	12.99±3.39[a]	0.05±0.01[b]	ND
	Methyl isovalerate	556-24-1	ND	ND	0.01±0.01[a]	ND	ND
	Citronellyl acetate	150-84-5	ND	ND	ND	ND	0.20±0.17[a]
	Neryl acetate	141-12-8	ND	ND	ND	ND	0.02±0.02[a]
	Phenethyl acetate	103-45-7	14.15±0.18[a]	0.01±0.02[c]	13.83±2.46[a]	0.00±0.00[c]	0.13±0.05[b]
	Isoamyl acetate	123-92-2	7.76±0.15[a]	0.00±0.00[b]	0.08±0.02[b]	0.00±0.00[b]	0.00±0.00[b]
	Neopentyl acetate	926-41-0	0.06±0.07[a]	ND	ND	0.03±0.03[a]	ND
	5-Decanolide	705-86-2	0.14±0.07[a]	0.05±0.01[b]	0.12±0.03[ab]	0.01±0.01[b]	0.03±0.02[b]
	Ethyl octanoate	106-32-1	0.03±0.00[a]	ND	0.01±0.00[a]	ND	ND
	Ethyl Hexanoate	123-66-0	0.86±1.50[a]	ND	0.01±0.01[a]	ND	ND
	Benzyl acetate	140-11-4	ND	ND	ND	ND	0.01±0.01[a]
Aldehydese	2-Methylbutanal	96-17-3	ND	ND	0.04±0.01[a]	ND	ND
	Benzaldehyde	100-52-7	0.02±0.04[a]	0.02±0.01[a]	0.02±0.01[a]	ND	ND
	Phenylacetaldehyde	122-78-1	0.07±0.13[b]	0.05±0.00[b]	5.64±1.23[a]	ND	ND
	2,6-Octadienal, 3,7-dimethyl-, (E)-	141-27-5	ND	ND	ND	ND	0.03±0.02[a]
	(Z)-3,7-dimethylocta-2,6-dienal	106-26-3	ND	ND	ND	ND	0.01±0.00[a]
	Hexanal	66-25-1	ND	ND	ND	0.02±0.02[a]	ND
	(E)-2-Octenal	2548-87-0	0.01±0.02[a]	ND	ND	ND	ND
Other substances	Undecane	1120-21-4	ND	ND	0.01±0.01[a]	0.0±0.01[a]	ND
	beta-Pinene	127-91-3	ND	ND	ND	ND	0.50±0.03[a]
	(+)-Limonene	5989-27-5	ND	ND	ND	ND	0.18±0.03[a]
	1,3,8-p-Menthatriene	18368-95-1	ND	ND	ND	ND	0.01±0.00[a]

(E)-Ocimene	3779-61-1	ND	ND	ND	ND	0.13±0.01[a]
γ-Terpinene	99-85-4	ND	ND	ND	ND	0.02±0.00[a]
M-cymene	535-77-3	ND	ND	ND	ND	0.04±0.03[a]
Terpinolene	586-62-9	ND	ND	ND	ND	0.05±0.00[a]
Methyleugenol	93-15-2	ND	ND	ND	ND	0.02±0.02[a]
(Z)-Ocimene	3338-55-4	ND	ND	ND	ND	0.21±0.02[a]

Note: Values are expressed as mean ± standard deviation (n=3). Different lowercase superscript letters in the same row indicate significant differences among groups (ANOVA, Duncan's multiple range test, $P < 0.05$). ND = Not Detected. The KMLC group is *K. marxianus* and *L. lactis* co-culture fermented milk system. The KM group is *K. marxianus* mono-culture fermented milk system; The LC group is *L. lactis* mono-culture fermented milk system; The C-PY group is commercial plain fermented milk, and C-RY is commercial rose-aroma fermented milk.

3 Results and discussion

Table S6

List of 37 key metabolites identified in KMLC fermented milk.

N0.	Compound	SuperClass	HMDB ID	Cluster_ID	KEGG_ID
1	Benzophenone	Benzenoids	HMDB0032049	Cluster 9	C06354
2	4-vinylphenol	Benzenoids	HMDB0004072	Cluster 3	C05627
3	Benzoic acid	Benzenoids	HMDB0001870	Cluster 3	C00180
4	Hippuric acid	Benzenoids	HMDB0000714	Cluster 9	C01586
5	Phosphoric acid	Homogeneous non-metal compounds	HMDB0000973	Cluster 9	C00009
6	2-hydroxy-3-methylbutyric acid	Lipids and lipid-like molecules	HMDB0000407	Cluster 3	NA
7	Cis,cis-muconic acid	Lipids and lipid-like molecules	HMDB0006331	Cluster 3	C02480
8	Lactulose	Lipids and lipid-like molecules	HMDB0000740	Cluster 7	C07064
9	Myristic acid	Lipids and lipid-like molecules	HMDB0000806	Cluster 9	C06424
10	Oleic acid	Lipids and lipid-like molecules	HMDB0000207	Cluster 9	C00712
11	Palmitic acid	Lipids and lipid-like molecules	HMDB0000220	Cluster 9	C00249
12	Uridine	Nucleosides, nucleotides, and analogues	HMDB0000296	Cluster 4	C00299
13	Creatinine	Organic acids and derivatives	HMDB0000562	Cluster 7	C00791
14	DL-proline	Organic acids and derivatives	HMDB0000162	Cluster 3	C00148
15	6-hydroxyhexanoate	Organic acids and derivatives	HMDB0012843	Cluster 3	C06103
16	Citrate	Organic acids and derivatives	HMDB0000094	Cluster 9	C00158
17	Dl-malic acid	Organic acids and derivatives	HMDB0000744	Cluster 9	C00497
18	L-(+)-lactic acid	Organic acids and derivatives	HMDB0000190	Cluster 3	C00186
19	Phenylalanine	Organic acids and derivatives	HMDB0000159	Cluster 7	C00187
20	Pyruvate	Organic acids and derivatives	HMDB0000243	Cluster 8	C00188
21	Acetylcholine	Organic nitrogen compounds	HMDB0000895	Cluster 5	C00189
22	Phosphocholine	Organic nitrogen compounds	HMDB0001565	Cluster 3	C00190
23	D-(+)-galactose	Organic oxygen compounds	HMDB0033704	Cluster 9	C00191
24	D-lactose	Organic oxygen compounds	HMDB0000186	Cluster 9	C00192
25	4-hydroxybenzaldehyde	Organic oxygen compounds	HMDB0011718	Cluster 3	C00193
26	N-acetylglucosamine	Organic oxygen compounds	HMDB0000215	Cluster 4	C00194
27	Pantothenate	Organic oxygen compounds	HMDB0000210	Cluster 2	C00195
28	Niacinamide	Organoheterocyclic compounds	HMDB0001406	Cluster 4	C00196
29	Adenine	Organoheterocyclic compounds	HMDB0000034	Cluster 9	C00197
30	Orotate	Organoheterocyclic compounds	HMDB0000226	Cluster 9	C00198
31	Purine	Organoheterocyclic compounds	HMDB0001366	Cluster 3	C00199
32	Uracil	Organoheterocyclic compounds	HMDB0000300	Cluster 2	C00200
33	Phenyllactic acid	Phenylpropanoids and polyketides	HMDB0000779	Cluster 3	C00201
34	Benzamidine	NA	NA	Cluster 2	C01784
35	Prophos	NA	NA	Cluster 9	C18687
36	Fenpropidin	Benzenoids	NA	Cluster 2	C18726
37	1-hydroxy-2-naphthoic acid	Benzenoids	NA	Cluster 3	C03203
